# Adverse effects of mefloquine for the treatment of uncomplicated malaria in Thailand: A pooled analysis of 19, 850 individual patients

**DOI:** 10.1371/journal.pone.0168780

**Published:** 2017-02-13

**Authors:** Sue J. Lee, Feiko O. ter Kuile, Ric N. Price, Christine Luxemburger, François Nosten

**Affiliations:** 1 Mahidol Oxford Research Unit, Mahidol University, Bangkok, Thailand; 2 Centre for Tropical Medicine and Global Health, Nuffield Department of Medicine, University of Oxford, Oxford, United Kingdom; 3 Department of Clinical Sciences, Liverpool School of Tropical Medicine, Liverpool, United Kingdom; 4 Global and Tropical Health Division, Menzies School of Health Research and Charles Darwin University, Darwin, Northern Territory, Australia; 5 Shoklo Malaria Research Unit, Mahidol-Oxford Tropical Medicine Research Unit, Faculty of Tropical Medicine, Mahidol University, Mae Sot, Thailand; Universidad Peruana Cayetano Heredia, PERU

## Abstract

Mefloquine (MQ) has been used for the treatment of malaria since the mid-1980s, first as monotherapy or as fixed combination with sulfadoxine-pyrimethamine (MSP) and since the mid-1990s in combination with artesunate. There is a renewed interested in MQ as part of a triple therapy for the treatment of multi-drug resistance *P*. *falciparum* malaria. The widespread use of MQ beyond south-East Asia has been constrained by reports of poor tolerability. Here we present the side effect profile of MQ for the treatment of uncomplicated malaria on the Thai-Myanmar/Cambodia borders. In total 19,850 patients received seven different regimens containing either 15 or 24–25 mg/kg of MQ, the latter given either as a single dose, or split over two or three days. The analysis focused on (predominantly) gastrointestinal and neuropsychiatric events as compared to the new fixed dose combination of MQ plus artesunate given as equal doses of 8 mg/kg MQ per day over three days. Gastrointestinal side effects were dose-dependent and associated with the severity of malaria symptoms. Serious neuropsychiatric side effects associated with MQ use were rare: for a single 25 mg/kg dose it was 11.9 per 10,000 treatments (95% confidence interval, CI, 4–285) vs. 7.8 (3–15) for the 15 mg/kg dose. The risk with 25 mg/kg was much higher when it was given as repeat dosing in patients who had failed treatment with 15 mg/kg MQ in the preceding month; (RR 6.57 (95% CI 1.33 to 32.4), p = 0.0077). MQ was best tolerated as 15 mg/kg or as 24 mg/kg when given over three days in combination with artesunate. We conclude that the tolerance of a single dose of MQ in the treatment of uncomplicated malaria is moderate, but can be improved by administering it as a split dose over three days.

## Introduction

Mefloquine (MQ) is a 4-aminoquinoline-methanol initially developed by the United States Department of Defense's Walter Reed Army Institute of Research in the mid-1970s (WR142,490; [1,2]). It is commercially available from Hoffmann-la-Roche (now Roche Holding A.G.) since the middle of the 1980s [3–5] (until 2009 in the USA; Lariam^®^) and is also widely sold as a generic. MQ was originally deployed as a monotherapy or in the fixed-dose combination MQ-sulfadoxine-pyrimethamine containing 250mg MQ, 500 mg sulfadoxine and 50 mg pyrimethamine (MSP, Fansimef^®^ [6,7]). However to counteract the emergence and spread of MQ-resistant parasites it was later combined with artesunate, an artemisinin derivative [8–14] and, since the mid-1990s this combination has been used extensively in South East Asia and South America.

The widespread deployment and uptake of MQ in other malaria-endemic regions has been constrained by reports of poor tolerability, particularly with respect to gastrointestinal [15], neuropsychiatric [16,17] and anecdotally ototoxic [18] adverse side effects. Several reviews have underlined differences in the prevalence of these adverse effects between various ethnic groups [19] as well treatment *versus* prophylactic usage [20].

The gastrointestinal side effects of MQ have been attributed to inhibition of pancreatic β-cell type-K_ATP_ channel Kir6.2/SUR1 [21]. Neuropsychiatric effects cover a spectrum of adverse events from mild anxiety to serious neurological and psychiatric adverse events, including psychosis, toxic encephalopathy, convulsions and 'acute brain syndrome' [22]. The exact molecular mechanism responsible for MQ neurological and psychiatric adverse events is poorly understood, but inhibition of cholinesterases [23,24], non-receptor tyrosine kinase 2 (Pyk2 [25]) and/or an interaction with adenosine A(2A) receptors [for the (-)-(R,S)-enantiomer in the MQ racemic mixture [26]] have all been implicated. The roles of the carboxylic acid metabolite or the enantiomers with regard to neuropsychiatric toxicity of MQ still need to be more fully elucidated [27,28]. A study found that the safety and tolerability of a single enantiomer (+)-MQ was not improved as compared with racemic MQ [29]. That study also reported the occurrence of dizziness, headaches, nausea and vomiting for various doses of racemic or (+)-MQ in healthy volunteers. Current estimates of the incidence of MQ serious adverse events (SAEs) have been derived mostly from retrospective studies and vary from 1 in 100–215 in Europeans to 1 in 1,754 treatments in Asians [13,30–33]. It is unclear whether the serious adverse reactions to MQ are dose-related [30].

**Fig 1:**
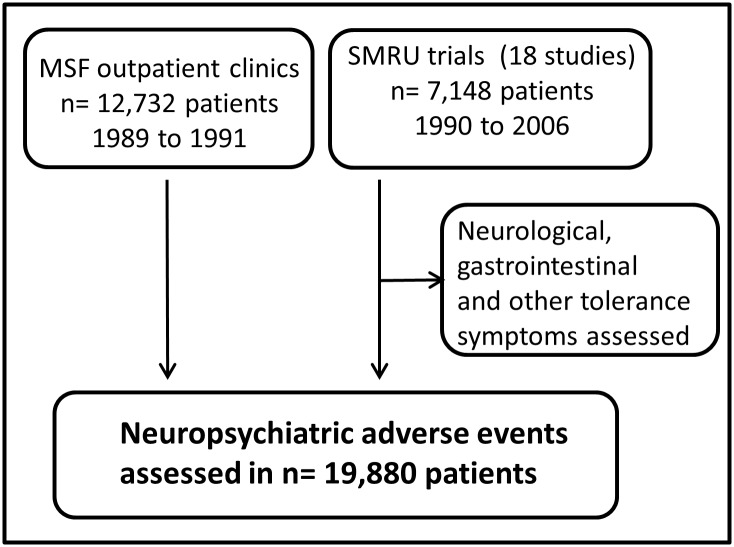
Overview of studies evaluated for neuropsychiatric adverse events. MSF: Médecins sans Frontières; SMRU: Shoklo Malaria Research Unit. These studies have been published: [13,14,34–48].

We present here an analysis from prospective clinical trials and retrospectively collated data of patients with uncomplicated malaria treated with low-dose (15 mg/kg) or standard-dose MQ (~25 mg/kg), either alone or in combination with artesunate, artemether or sulfadoxine-pyrimethamine, in regions around the Thai-Myanmar/Cambodia borders (Fig 1). The aim was to define the risk of gastrointestinal and neuropsychiatric side effects, to explore the association of demographic and clinical factors on tolerability, and to determine if splitting the dose of MQ over three days decreased the risk of adverse events.

## Methods

Our analysis is divided into two parts. The first part reviews the tolerance of MQ in 7,148 patients enrolled in 18 clinical trials and treated with 15 or 25 mg/kg MQ alone or in combination with artesunate, artemether or sulfadoxine-pyrimethamine, over a 17-year period (1990–2006) on the Thai-Myanmar border. The second part of the analysis on neuropsychiatric adverse events includes an additional 12,732 patients with uncomplicated malaria treated with 15 or 25 mg/kg of MQ alone or in combination with artesunate or sulfadoxine-pyrimethamine from 1989 to 1991 in out-patient clinics along the Thai-Myanmar/Cambodia borders as part of routine patient care by Médecins Sans Frontières (MSF) and followed by passive case detection.

### Patients and treatments

The clinical studies included in the first analysis were conducted to optimize the treatment of uncomplicated malaria against the backdrop of increasingly drug-resistant malaria. The detailed methods of these trials have been published in the respective reports (see Legend to Fig 1). Briefly, the trials recruited patients with uncomplicated malaria either infected with *P*. *falciparum* alone or co-infected with *P*. *vivax*, confirmed by microscopy, provided they or their guardian gave written informed consent. The exclusion criteria included patients presenting with signs and symptoms of severe infection, hyperparasitaemia (>4% infected red blood cells), children and infants weighing less than 5 kg, pregnant women and after 1994, patients who had been treated with MQ in the preceding two months. MQ was used either as monotherapy or in combination with an artemisinin derivative (with the exception of one study which was in combination with SP; Table 1). Treatment was administered under supervision. If vomiting occurred within 30 minutes of drug administration the full dose was re-administered. Patients who vomited between 30–60 minutes after treatment were re-administered half the initial dose. No re-treatment was given for vomiting after 60 minutes or later. At each visit, a blood slide was prepared and each patient was seen daily until parasite-negative, then weekly for 4–9 weeks. On admission and at each follow-up visit a basic clinical examination was performed, a history of clinical illness taken and a standard side-effects questionnaire completed.

**Table 1:** The timing and total dose of MQ (mefloquine) and artesunate from 18 SMRU studies.

Dosing regimen	MQ dose (mg/kg)	Combined/Split	Day of MQ admin.	Partner drug	Partner drug admin.	ART day = 0 (mg/kg)	ART day = 1 (mg/kg)	ART day = 2 (mg/kg)	Total ART dose (mg/kg)
1	15	Combined	D0	SP	D0				
1	15	Combined	D0	--	--				
1	15	Combined	D0	ART	D0	10			10
2	25	Combined	D0	--	--				
2	25	Combined	D0	Art/Am	D0 (D1/D2)	4			4
3	25	Combined	D1	Art	D0/D1/D2	4	4	4	12
3	25	Combined	D1	--	--				
4	25	Combined	D2	Art/Am	D0 D1 D2	4	4	4	12
5	25	Split (15/10)	D1/D2	ART	D0 D1 D2	4	4	4	12
6	25	Split (15/10)	D0/D1	--	--				
7	25	Split (8+8+8)	D0/D1/D2	ART	D0/D1/D2	4	4	4	12

MQ was given combined in a single administration or split in two or three doses; admin.: administration; ART: artesunate; Art/Am: artesunate or artemether; MQ: mefloquine; SP: sulfadoxine/pyrimethamine.

The second analysis to quantify the risk of serious neurological or psychiatric complications included an additional 12,732 patients with uncomplicated *P*. *falciparum* treated at MSF clinics from 1989 to 1991. Patients from these MSF clinics were not routinely followed-up. Serious neurological or psychiatric complications were defined as any event involving the Central Nervous System (CNS) and requiring medical attention. These included acute psychosis, delusions, hallucinations, anxiety neuroses, major disorders of affect, disturbed consciousness, and seizures. A preliminary analysis of serious neuropsychiatric events observed in the MSF clinics was published previously [35].

The history of previous antimalarial treatment was documented from the MSF and SMRU records. For each patient, the presence or absence of behavioral changes was recorded at each visit. All cases suspected of serious neuropsychiatric reactions by the study nurse or reported by the family were assessed by a physician at the MSF or SMRU clinics (who were the only providers of healthcare in the area at the time), or otherwise visited at home by the treating physician. The number of events and exact denominator were obtained from standardized monthly malaria treatment records. No details on associated risk factors were available from these records.

All the studies were approved by the relevant ethics and institutional review committees: the Faculty of Tropical Medicine Ethical Committee at Mahidol University (Bangkok, Thailand) and the Oxford Tropical Research Ethics Committee (OXTREC), University of Oxford, UK.

### Tolerance evaluation: Frequency of adverse events

Gastrointestinal events reported included: vomiting, nausea, anorexia, abdominal pain, and diarrhea. Neuropsychiatric events included: headache, dizziness, rigors, ataxia, sleep disturbances, tremors, and palpitations. All other signs and symptoms were classified as ‘other’. The patients were assessed for signs and asked about specific symptoms experienced during the last 24 h. Therefore a report on Day1 reflects the occurrence of this event within approximately the first 24 hours after the start of treatment, a report on Day 2 reflects an occurrence during the 2^nd^ period of 24 hours, *etc*.

‘Early vomiting’ was defined as vomiting within 1 hour after receiving a dose of MQ, and ‘late vomiting’ as occurring in the subsequent 23 hours. Fever was defined by an axillary temperature >37.5°C, an oral temperature >38°C and/or patients reporting fever in the preceding 48 hours.

Neurological events (psychiatric and others) were documented in the early SMRU trials up to 2001 inclusive, focusing on the presence of ataxia (Romberg test [49,50], rapid alternate movements), mobility (tandem walk), fine mobility (picking a tablet), and oculo-vestibular abnormalities (nystagmus) [15]. However in the absence of any noticeable effects, these events were not reported in subsequent trials. Data on other neuropsychiatric events, such as tremors, nightmares or sleep disturbances, impaired hearing and dizziness continued to be collected throughout the study period.

### Statistical analysis

Data were summarized using frequency (n/N, %), geometric means (95% confidence interval [CI]) or median (inter-quartile range [IQR]), as appropriate. The frequency of early vomiting was described overall for an individual and also according to episodes, in which case a patient vomiting on multiple days could be represented more than once. The risk of early vomiting was calculated by treatment regimen and plotted over the first three days. For dizziness, nausea, late vomiting and anorexia, frequencies were plotted up to day 28. Patients younger than 5 years old were excluded from analyses of dizziness and nausea.

The risk of adverse events was assessed according to the MQ regimen administered, using as the reference group the new fixed dose combination of MQ plus artesunate, which is given as equal doses of 8 mg/kg MQ per day over three days (total 24 mg/kg MQ). The six other MQ-containing dosing regimens included: single 15 mg/kg dose received on day 0, single 25 mg/kg dose received on day 0, single 25 mg/kg dose received on day 1, single 25 mg/kg dose received on day 2, 25 mg/kg as split dose (15/10), given either as 15 mg/kg on day 0 and 10 mg/kg on day 1, or as 15 mg/kg on day 1 and 10 mg/kg on day 2 (all the latter patients received artesunate on day 0; Table 1).

The risk rate was calculated as the number of serious adverse events divided by the total number of patients seen over the same period with exact confidence intervals. Relative risks and confidence intervals were estimated using Poisson regression with robust error variances [51]. The median time to resolution of symptoms present at enrolment was calculated by survival analysis using the Kaplan-Meier method [52], the event being the resolution of the symptom within seven days. Patients with a symptom for whom data were missing were censored from the last day of a valid observation.

Statistical data analysis was done using STATA, v13 (StataCorp, College Station, TX, USA).

## Results

### Baseline characteristics of the patients in the clinical trials

For the analysis of MQ tolerance, a total of 7,148 patients were available from 18 SMRU trials conducted between 1990 and 2006 (Fig 1). In the earlier studies (1990–1992, n = 2,265), 40% of patients (n = 904) received a single 15 mg base/kg dose. The remaining 6,244 patients were treated with 24 or 25 mg/kg of MQ (henceforth referred to as 25 mg/kg) administered either as a single dose (n = 3,212) or as a split dose of 15 mg/kg followed by 10 mg/kg (10+15, n = 2,305), or three 8-mg/kg doses over three days (8+8+8, n = 727; Table 1).

Patient baseline characteristics at presentation varied significantly over the 16 year study period (all p < 0.001 for variation by year, data not shown). Overall, the median (IQR) age of the study participants was 14 years (8–25) years. Nearly two thirds of the participants (60%) were male and the majority presented with P. *falciparum* mono-infection at admission (86%, Table 2).

**Table 2:** Trial participant baseline characteristics.

Parameter	Total	Summary statistic
Age in years	7,148	14 (0.24, 95)
Patients younger than 5 years		750 (10.5%)
Aged 5–14 years		2,901 (40.6%)
Aged 15 years and older		3,497 (48.9%)
Male	7,148	4,309 (60.3%)
Geometric mean parasitaemia microl^-1^ (CI)	7,064	4,179 (3,966–4,403)
Temperature °C	6,599	38.1 (1.11)
Fever at presentation*	6,636	5,372 (81.0%)
Baseline HCT %	5,633	36.1 (5.82)
Species at presentation	6,480	
*P*. *falciparum*		5,547 (85.6%)
*P*. *vivax*		1 (0.02%)
Mixed (*Pf* with *Pv*)		929 (14.3%)
Mixed (*Pf* with *Po* or *Pm)*		3 (0.05%)

Numbers are mean (SD), Median (min, max) or frequency (%), unless otherwise specified. CI: 95% confidence interval; HCT: hematocrit; *Pf* with *Po* or *Pm*: *Plasmodium falciparum* with *P*. *ovale* and *P*. *malariae*; SD: standard deviation.

*Defined as temperature >37.5°C or reported fever in past 48 hours.

### Overview of adverse events

Table 3 lists 26 adverse events that occurred within the first 28 days of follow-up. The most common neuropsychiatric events on admission were headache (82%), dizziness (47%), and sleep disturbance (22%). The most common gastrointestinal complaints at admission were anorexia (62%), nausea (35%), vomiting (23%), and abdominal pain (21%). The frequency of all of these adverse effects reduced over time, in parallel with the resolution of malaria-associated symptoms.

**Table 3:** Frequency of adverse events.

		(Baseline) Day 0		Day 1		Day 2		Day 3		Day 7		Day 28	
	Adverse event		%		%		%		%		%		%
**Gastrointestinal**	Late vomiting*	1,473/6,462	22.8	976/6,006	16.3	362/5,342	6.78	141/2,532	5.57	54/4,537	1.19	33/3,877	0.85
	Nausea	2,099/5,949	35.3	1,641/5,528	29.7	932/4,959	18.8	347/2,331	14.9	166/4,259	3.9	68/3676	1.85
	Anorexia	4,006/6,448	62.1	3,490/5,993	58.2	2204/5,339	41.3	853/2,526	33.8	628/4,530	13.9	232/3,873	5.99
	Abdominal pain	1,277/6,034	21.2	878/5,607	15.7	522/4,985	10.5	189/2,254	8.39	159/4,213	3.77	97/3,625	2.68
	Diarrhea	96/3,105	3.09	113/3,013	3.75	81/2,900	2.79	21/1,269	1.65	26/2,469	1.05	22/2,227	0.99
**Neuropsychiatric**	Headache	4,943/6,033	81.9	3,141/5,585	56.2	1,525/5,000	30.5	561/2,364	23.7	534/4,265	12.5	333/3,654	9.11
	Dizziness	2,622/5,549	47.2	2,417/5,154	46.9	1,839/4,638	39.7	868/2,115	41	832/3,963	21	237/3,415	6.94
	Rigors	923/4,930	18.7	579/4,583	12.6	260/3,964	6.56	102/1,537	6.64	111/3,244	3.42	88/2,726	3.23
	Romberg test	12/1,657	0.72	13/1,224	1.06	8/1,194	0.67	2/152	1.32	3/1,343	0.22	1/1,062	0.09
	Tandem walk	16/1,705	0.94	11/1,272	0.86	11/1,241	0.89	5/201	2.49	4/1,345	0.3	2/1,112	0.18
	Rapid alt. mvts.	2/499	0.4	1/267	0.37	0/274	0	0/39	0	0/408	0	0/357	0
	Pick a tablet	4/1,710	0.23	4/1,276	0.31	2/1,245	0.16	0/201	0	2/1,347	0.15	0/1,113	0
	Ataxia	0/50	0	n.e.	n.e.	n.e.	n.e.	n.e.	n.e.	n.e.	n.e.	n.e.	n.e.
	Sleep disturb.	567/2,597	21.8	577/2,514	23	477/2,439	19.6	195/1,073	18.2	223/2,101	10.6	39/1,931	2.02
	Tremors	32/1,346	2.4	26/1,326	1.96	18/1,307	1.38	10/455	2.2	16/1,160	1.38	3/1,108	0.27
	Hearing	91/2,646	3.44	82/2,208	3.71	63/2,164	2.91	28/904	3.1	22/2,151	1.02	10/1,906	0.52
	Nystagmus	28/2,025	1.38	19/1,594	1.19	20/1,566	1.28	12/511	2.35	11/1,659	0.66	7/1,426	0.49
**Other**	Rash	63/2,223	2.83	56/2,187	2.56	47/2,156	2.18	17/837	2.03	24/1,878	1.28	16/1,755	0.91
	Urticaria	3/893	0.34	2/887	0.23	3/879	0.34	1/430	0.23	1/741	0.13	0/672	0
	Fever	5,374/6,639	81	2701/5,502	49.1	735/4,845	15.2	199/2,024	9.83	171/4,040	4.23	180/3,382	5.32
	Weakness	2,081/3,433	60.6	1617/3,074	52.6	972/2,587	37.6	510/1,415	36	421/2,232	18.9	153/1,888	8.1
	Muscle/joint pain	3,112/5,275	59	1,866/4,937	37.8	901/4,422	20.4	307/1,845	16.6	302/3,694	8.18	211/3,174	6.65
	Palpitations	484/2,081	23.3	412/2,064	20	343/2,041	16.8	153/804	19	161/1,772	9.09	36/1,676	2.15
	Enlarged spleen	1453/6,288	23.1	n.e.	n.e.	n.e.	n.e.	n.e.	n.e.	n.e.	n.e.	n.e.	n.e.
	Enlarged liver	1001/6,295	15.9	n.e.	n.e.	n.e.	n.e.	n.e.	n.e.	n.e.	n.e.	n.e.	n.e.
	Fatigue	761/2,181	34.9	647/2,158	30	432/2,127	20.3	122/828	14.7	180/1,843	9.77	69/1,728	3.99

*vomiting occurring at least one hour after MQ administration; n.e.: not evaluated; Rapid alt. mvts.: rapid alternate movements.

### Early vomiting

**Fig 2:**
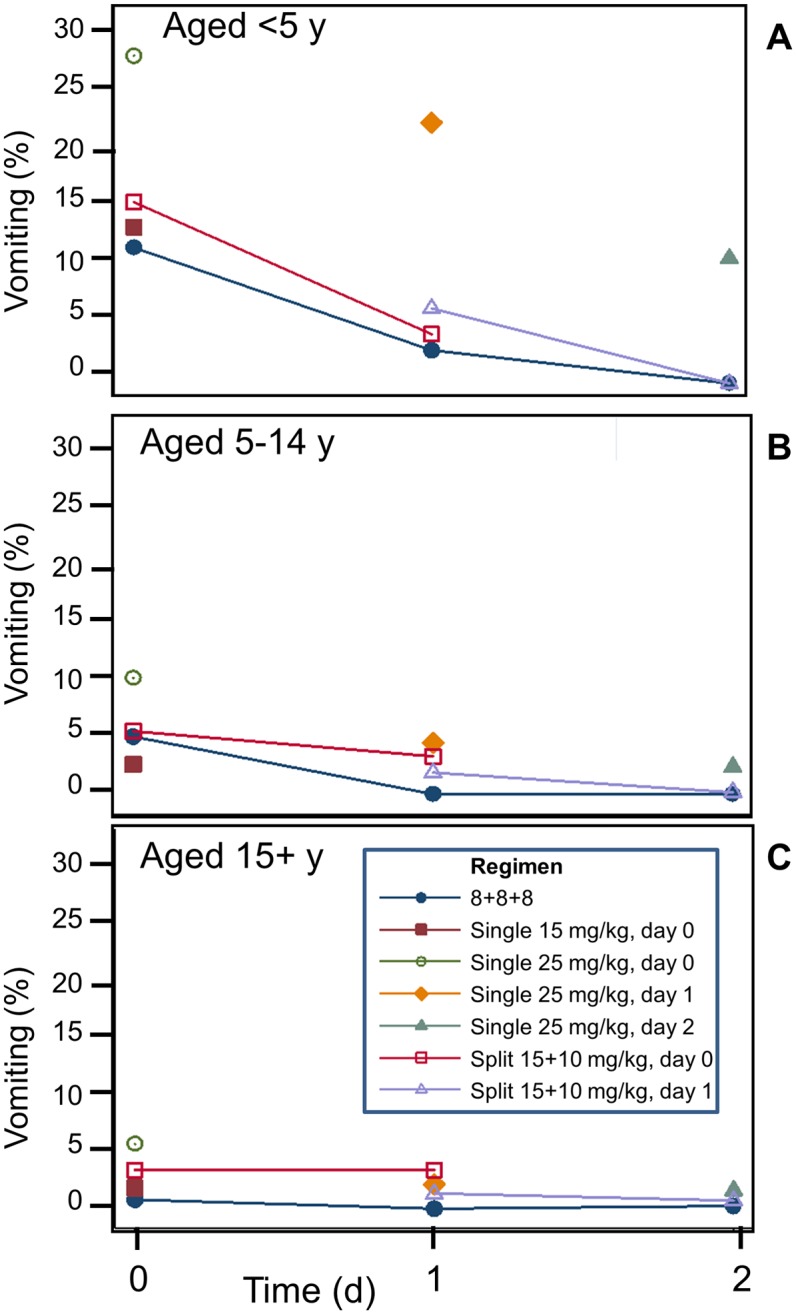
Vomiting frequency (by episode) directly (<1 h) after mefloquine (MQ) administration. Frequencies are by age group (**A-C**) and day of treatment.

**Fig 3:**
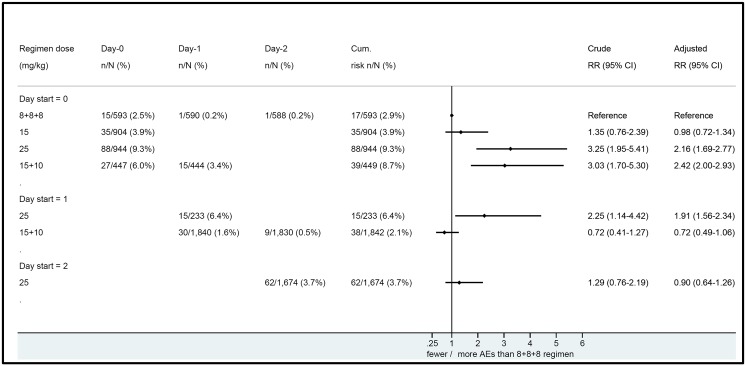
Forest plot of adjusted cumulative risk of early vomiting compared with the 8+8+8 treatment group (8 mg/kg MQ on three consecutive days). Adjusted analyses were stratified by year and included covariates for age, sex, baseline hematocrit, fever at admission, baseline [log] parasitaemia and vomiting at admission; more specifically to the latter, the occurrence of early vomiting on day 0 for day 1 risk estimates and the occurrence of early vomiting on day 1 for day 2 risk estimates. Cumulative risks were defined as any vomiting on any day within 1 hour of MQ treatment. The cumulative frequency for 8+8+8 was 17/593 (for daily frequencies, see S1 Table).

The frequency of early vomiting was highest in young children: 14% (106/735) for children younger than 5y old, compared to 3.8% (108/2,834) in children aged 5-14y and 2.5% (81/3,310) in older children and adults (p<0.001). The age-associated patterns in risk of vomiting were similar across treatment groups (Fig 2 and Table 4). The risk of early vomiting was highest on the first days of treatment and with a higher daily MQ dose. The 8+8+8 regimen was associated with significantly less early vomiting as compared to most other regimens (Fig 3). This adverse effect of MQ is dose-related and more frequent with a single dose of 25 mg/kg than with a single dose of 15 mg/kg. The adjusted cumulative risk for patients vomiting any of their MQ doses over three days showed a similar trend (higher risk with increasing dose of MQ and in the first days of treatment). The 8+8+8 regimen provided the best tolerability profile, and this remained so after stratifying by year (S1 Table).

**Table 4:** Unadjusted relative risks of any early vomiting (95% CI), by age group.

	Age < 5y		Age 5–14 y		Age >15 y	
Regimen	Freq. (%)	RR (CI)	Freq. (%)	RR (CI)	Freq. (%)	RR (CI)
Single 15 mg/kg, d0	19/144 (13.2%)	0.92 (0.37,2.31)	9/360 (2.50%)	0.52 (0.20,1.31)	7/400 (1.75%)	1.72 (0.51,5.82)
Single 25 mg/kg, d0	27/98 (27.6%)	1.39 (0.90,2.15)	34/351 (9.69%)	1.41 (0.97,2.05)	27/501 (5.39%)	2.30 (1.36,3.87)
Single 25 mg/kg, d1	10/41 (24.4%)	1.20 (0.86,1.66)	4/93 (4.30%)	0.96 (0.65,1.42)	2/99 (2.02%)	1.26 (0.72,2.20)
Single 25 mg/kg, d2	28/266 (10.5%)	0.93 (0.74,1.16)	26/1125 (2.31%)	0.83 (0.68,1.01)	8/517 (1.55%)	1.11 (0.82,1.50)
Split 15/10 mg/kg, start d0	12/72 (16.7%)	1.03 (0.85,1.25)	16/189 (8.47%)	1.12 (0.95,1.32)	11/188 (5.85%)	1.42 (1.13,1.78)
Split 15/10 mg/kg, start d1	5/79 (6.33%)	0.87 (0.72,1.06)	11/551 (2.00%)	0.86 (0.74,1.00)	22/1213 (1.81%)	1.1 (0.92,1.31)
3-day 8+8+8	5/35 (14.3%)	Ref.	8/165 (4.85%)	Ref.	4/392 (1.02%)	Ref.

All treatments are compared with the ‘8+8+8’ MQ regimen (8 mg/kg MQ administered on three subsequent days). Early vomiting was defined as vomiting occurring within one hour of MQ treatment. The unit of analysis was by patient, *i*.*e*., if a patient vomited on all three days of treatment with 8+8+8, this was counted only once. Risks and confidence intervals were estimated using Poisson regression with robust error variances.

### Late vomiting, anorexia, nausea and dizziness

The occurrence of symptoms at enrolment was associated with an increased frequency of subsequent side effects (S1 Fig). The frequencies of late vomiting, anorexia, nausea and dizziness were lower in patients who received the 8+8+8 treatment regimen (S2 Fig). In older children and adults, most symptoms had resolved by Day 28, with the exception of dizziness, which persisted in some patients, particularly in those who had already experienced dizziness upon admission. The median (IQR) time to resolution of symptoms was 1 or 2 days (1–3 days).

### Serious neuropsychiatric adverse events

The risk of serious neuropsychiatric adverse events was assessed in 12,732 patients from the MSF outpatient clinics and in 7,148 patients from the SMRU trials.

Between 1989 and 1991 at the MSF clinics, there were seven serious neuropsychiatric events (five on 15 mg/kg MQ and two on 25 mg/kg MQ) reported from a total of 9,468 patients who received 15 mg/kg of MQ and 3,264 who received M25 treatment, respectively (Table 5). Detailed of 4 available case reports for these events are presented in S2 Table.

**Table 5:** Serious neuropsychiatric events associated with MQ treatment.

	Nr. of cases/nr. of patients				
Regimen	SMRU trials	MSF clinics^a^	Pooled	Risk (95% CI)	Risk ratio (95% CI)
8+8+8	0/727	-	0/727	-	
Single, 15 mg/kg, d0	3/904*	5/9,468	8/10,372	7.71/10,000 (3.33–15.2)	Ref.
Single, 25 mg/kg, d0	3/952**	2/3,264	5/4,216	11.9/10,000 (385–27.7)	1.54 (0.50–4.70)
Single, 25 mg/kg, d1	0/350	-	0/350	-	
Single, 25 mg/kg, d2	2/1,910^†^	-	2/1,910	10.5/10,000 (1.27–37.8)	1.36 (0.29–6.39)
15/10 mg/kg, start d0	0/453	-	0/453	-	
15/10 mg/kg, start d1	0/1,852	-	0/1,852	-	
TOTAL	8/7,148	7/12,732	15/19,880	7.55/10,000 (4.22–12.4)	-

^a^ Derived with permission from Luxemburger *et al*., [35], after exclusion of 1,236 patients overlapping with those from the SMRU malaria research trials in the first column. MSF: Médecins Sans Frontières

* Two SAEs in this group (1 female, 1 male) occurred after re-treatment with 25 mg/kg MQ for recrudescence.

** Two patients in this group (1 female, 1 male) had a history of major neuropsychiatric disorders.

^†^ One patient in this group (female) had a history of major neuropsychiatric disorders.

In the SMRU trials, there were a total of eight serious neuropsychiatric adverse events reported. Six (75%) events occurred in women. Two of these SAEs were documented amongst 345 patients who received 25 mg/kg of MQ within two months of a previous treatment with MQ, while the remaining 6 SAEs occurred in 6,803 patients who had not received MQ in the previous two months (RR 6.57 (95% CI 1.33 to 32.4), p = 0.0077). Three of the eight patients (37.5%) had reported a history of previous neuropsychiatric disorders. While the numbers are low (7 and 8), there is evidence that overall frequencies in the incidence of EAs at the SMRU and MSF clinics differ (0.5 *vs* 1.1/1,000 treatments). If real, this might point to the importance of passive *vs* active AE monitoring.

The overall risk of neuropsychiatric events was 7.6/10,000 (95% CI 4 to 12); 7.7/10,000 among patients who received 15 mg/kg and 10.5 to 12/10,000 among patients who received 25 mg/kg MQ (Table 5).

### Deaths

Three patients died during the follow up period. A 34 year old male with a history of opium addiction died one month after receiving MQ (15mg/kg) and 3 days of artesunate (2 mg/kg/d). He had a negative malaria smear by Day 2 and was hospitalized with pneumonia and cachexia 4 days before death. A 13 year old male died at home on Day 5 after 8+8+8 MQ treatment with an acute surgical abdomen. The third death was a land mine accident in a 15 y old male on Day 10 after 15 mg/kg MQ. None of these deaths were considered to be related to the treatment.

## Discussion

Our analysis demonstrates that, overall, the main adverse effects of MQ are dose-related and more frequent with a single dose of 25 mg/kg than with a single dose of 15 mg/kg, as reported previously. Our analysis also shows that splitting the 25 mg/kg dose over two days reduced this difference and importantly, when 25 mg/kg was given as 8 mg/kg per day over three consecutive days, the tolerance was similar to that observed with a 15 mg/kg single dose. The frequency of adverse events was also lower in the group that received artesunate alone on Day 0 and when MQ treatment commenced as fever and other symptoms abated. The influence of dose and timing was most apparent in young children (<5 years) and those 5–15 years, although dizziness was more prevalent and longer-lasting in adults. Overall, the 8+8+8 regimen provided the best tolerability compared to the other regimens and this was apparent for early vomiting, nausea and dizziness, although not for late vomiting and anorexia in the youngest age group.

Neuropsychiatric reactions associated with MQ treatment are more difficult to assess since such events are rare, even within such a large dataset as the one in the current pooled analysis. Furthermore malaria itself can be associated with neuropsychiatric complications [53], though these are mostly seen in cerebral malaria caused by *Plasmodium falciparum*, and only patients with uncomplicated malaria were enrolled in this analysis. The neuropsychiatric events were assessed by a physician at every visit. We only recorded serious adverse event; these were very obvious and were those spontaneously reported. It is possible that mild events were missed. A causal relationship for neuropsychiatric reactions was suggested by the strong temporal relationship with MQ treatment. In all but one patient, these events started within the first few days after drug administration; in the remaining patient the event occurred on day 27. When patients were re-treated with 25 mg/kg MQ within one month of initial treatment with 15 mg/kg the risk of serious events was almost seven-fold higher than with primary 25 mg/kg MQ treatment. Two of the four patients with serious psychiatric reactions had a history of psychiatric disorders, reinforcing the contraindication of MQ in such patients. However, they also occurred in patients with no prior history, as well as those who had previously tolerated MQ treatment well.

A limitation of our study is that we only looked at therapeutic use of MQ, in a well-defined patient cohort. We deliberately chose to analyze the (large) patient cohorts presented in this study separately from other studies because they are from the same region and were evaluated using common criteria. One earlier chemoprophylaxis study for MQ reported a 7.9% occurrence of AEs among 420 volunteers, but none serious [54]. Older studies had estimated the risk for neuropsychiatric SAEs at one per 10,600 [55] or one per 13,000 [31], which are about ten-fold lower than what we report here. Another caveat is that somewhat different inclusion/exclusion criteria existed for the trials whose results we have combined. However, most of these (early pregnancy, concomitant use of other medicines) are shared. Among our study's strength are the involvement of a relatively homogenous population that is closely followed medically, and the small number of clinical centers.

The patient population lived in camps for displaced persons on the Thai-Myanmar border, providing an opportunity to closely monitor rates of serious events. The immediate access to health care in this relatively confined area and its high utilization facilitated good follow-up. MSF and the Shoklo Malaria Research Unit (SMRU) were the only suppliers of antimalarials at the time of these studies within these camps and external drug sources were difficult to obtain for the displaced persons. We are thus confident that we have an accurate estimate of the total number of treatments given per period. Although minor, transient changes in behavior and sleep disturbances may go unnoticed in this setting, serious, potentially life-threatening events, such as acute psychosis, convulsions and impaired consciousness would almost certainly be brought to the attention of these health services.

The authors are aware of additional clinical studies where the safety of mefloquine was assessed. Previous studies have attempted to correlate the MQ blood drug concentrations and the risk of serious [30], or non-serious [15] MQ-related events, but the results have been inconclusive [47]. We decided against their inclusion for this analysis because of the different methodologies and populations that were involved.

Of interest is that the tolerance was best in patients treated with the 8+8+8 regimen, which of all the 25 mg/kg regimens is best absorbed and results in higher drug concentrations of MQ than achieved with a single dose or split dose of 25 mg/kg. This suggests that peak concentration *per se* is not a main determinant of tolerance but that perhaps the rate at which peak concentrations are reached is the better predictor of tolerance. The recent WHO malaria treatment guidelines [56] recommend against the use of MQ within 60 days of a previous exposure because of the risk of neuropsychiatric toxicity. The increased risk of neuropsychiatric toxicity in enrolled patients re-exposed to a second treatment course within a month also suggests priming of the CNS, since SAEs were not experienced upon first exposure to MQ. MQ has extensive tissue distribution [57] and it is not known whether patients with neuropsychiatric reactions have particularly high cerebrospinal concentrations, nor whether clearance of MQ varies by organ. If clearance from the CNS were particularly slow then patients who had received MQ previously (≤ 1 month) might still have a residual CNS concentration. These data suggest that MQ should not be used as a retreatment drug for MQ failures, also because little clinical benefit is gained from administration of MQ as second-line treatment of MQ failures. Our analysis includes data from patients recruited over a 16-year period during which there were significant variations in the trial designs, baseline characteristics and data acquisition. Specifically, reporting of adverse events may have differed between younger patients (the majority cohort seen in the early years) and young adults (more frequently seen in later years). Nevertheless our analysis sheds light both on the common MQ-associated complications of gastrointestinal nature and the more serious but less frequent neuropsychiatric symptoms.

As for many other infectious diseases, the speed at which malaria parasites develop drug resistance rapidly outpaces the rate at which new medicines are discovered; rather than eliminating the disease there is a very real danger that it will instead make another comeback, as happened in the second half of the 20^th^ century with the loss of the second generation of antimalarials [58]. Resistance against the latest generation of malaria drugs, the artemisinins, is spreading in southeast Asia [59], and its emergence in Africa could be catastrophic. There is increasing biological understanding of the costs of resistance to the pathogen. For instance, it was recently found that resistance against atovaquone results in a failure of the parasite to be transmitted by mosquitoes [60], and also of resistance incompatibilities. While waiting for new drugs [61], this suggests strategies whereby 'older' antimalarials are rotated, or administered as two- or three drug regimens (as is routine in other infectious diseases such as HIV/AIDS and tuberculosis). This in turn requires that we update the safety and efficacy data for these drugs as more experience is gained from them. The safety of MQ is somewhat controversial yet we feel the drug still has an important role to play in managing disease. A recent study in African children has confirmed to safety of ASMQ and this will help with the deployment of this useful combination in Africa [62].

## Conclusions

In conclusion, our data confirm earlier findings that gastrointestinal and neuropsychiatric adverse events following MQ are dose-related, but that 25 mg/kg MQ is much better tolerated when the total dose is given over three days instead of two days, or as a single dose. We recommend administering MQ as a split dose with strict adherence to the contraindication of MQ in those with a history of psychiatric illness or re-treatment with MQ within 2 months of prior MQ therapy as per WHO guidelines. Our analysis highlights the need for the continued monitoring of drugs as their use expands, to improve our understanding of adverse effects so that there their usage can be optimized to maximize tolerability without compromising efficacy.

## Supporting information

S1 Fig Frequency of late vomiting, anorexia, dizziness, and nausea each day, by age group and by treatment regimen, and by presence of the indicated symptom at admission. (DOCX)

S2 Fig Relative risks (95% confidence interval) of dizziness, nausea, anorexia, or late vomiting by treatment regimen, as compared to the 8+8+8 regimen. (DOCX)

S1 Table Frequency (%) of early vomiting and relative risk (RR, unadjusted and ARR, adjusted), by day and by treatment regimen. (DOCX)

S2 Table Case reports for serious neuropsychiatric adverse events. (DOCX)
