# Supplementary material for: Adverse effects of mefloquine for the treatment of uncomplicated malaria in Thailand: A pooled analysis of 19, 850 individual patients
Source: PLoS One. 2017 Feb 13;12(2):e0168780. doi: 10.1371/journal.pone.0168780 (PMC5305067; doi:10.1371/journal.pone.0168780)
Supplement: S1 Fig — (DOCX) [file pone.0168780.s001.docx]

**S1 Fig**: **Frequency of late vomiting, anorexia, dizziness, and nausea each day, by age group and by treatment regimen, and by presence of the indicated symptom at admission.** The unit of analysis was an episode, *i.e*., if a patient reported dizziness on days 1, 2 and 7, this was counted three times, once on each specified day. Denominators were the number of patients with non-missing symptom data for that treatment on the specified day. The value at day 28 is an average of the values from day 28 to day 63. Children younger than 5 years old were excluded from the analysis for dizziness and nausea. “NO at admission” shows data for the subset of patients who did not report the specified symptom at admission.
