# Supplementary material for: Adverse effects of mefloquine for the treatment of uncomplicated malaria in Thailand: A pooled analysis of 19, 850 individual patients
Source: PLoS One. 2017 Feb 13;12(2):e0168780. doi: 10.1371/journal.pone.0168780 (PMC5305067; doi:10.1371/journal.pone.0168780)

**S2 Fig**: **Relative risks (95% confidence interval) of (A) dizziness, (B) nausea, (C) anorexia, (D) late vomiting by treatment regimen, as compared to the 8/8/8 regimen.**

**
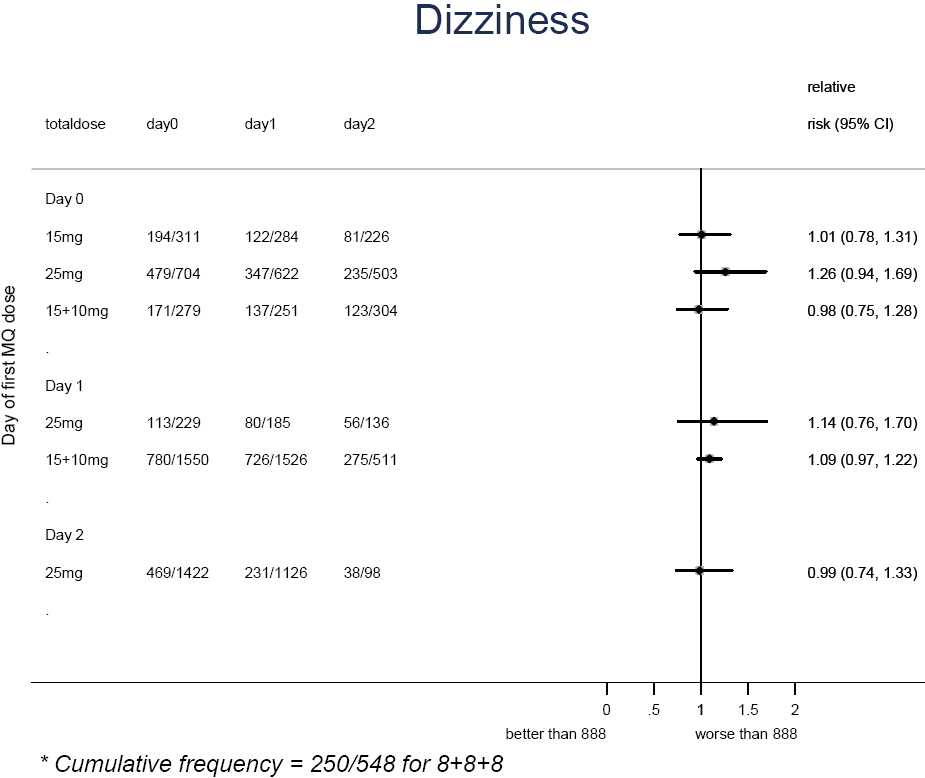
**


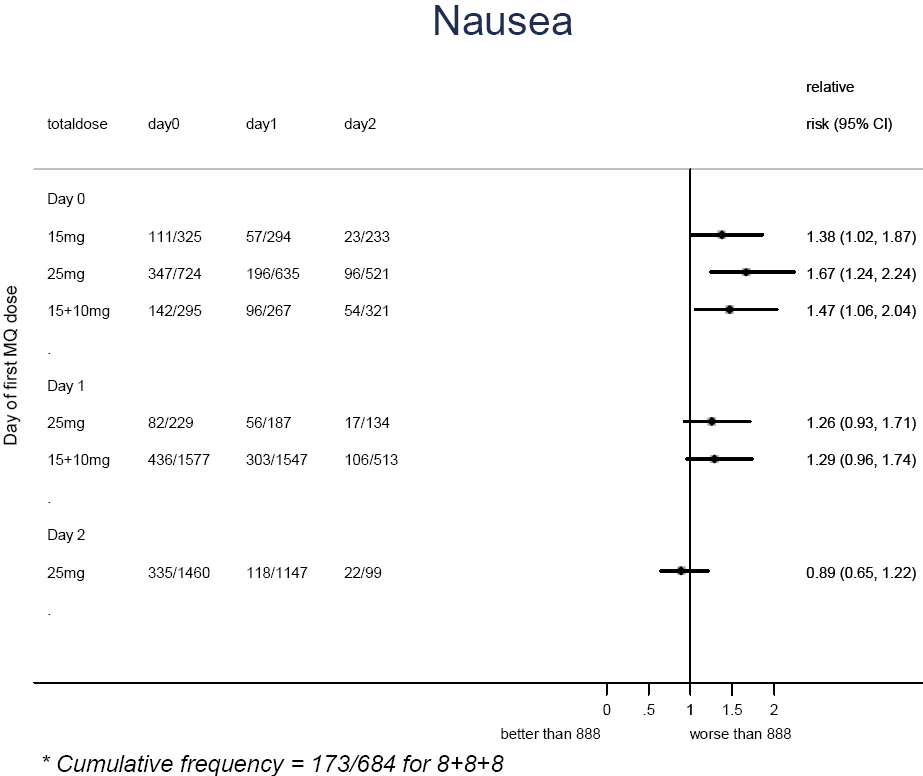


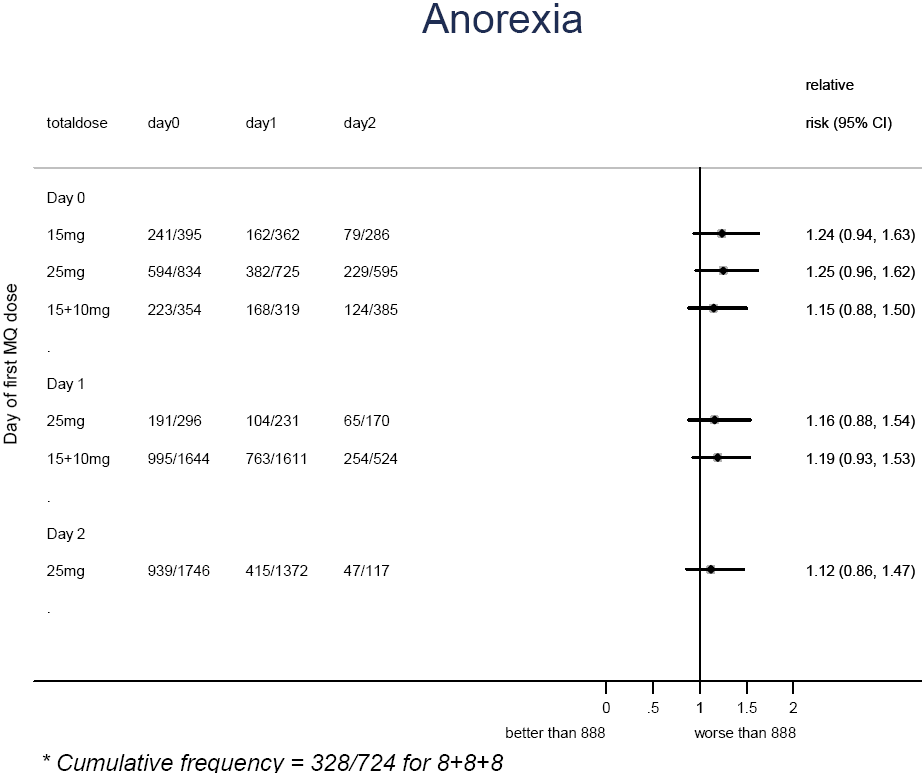


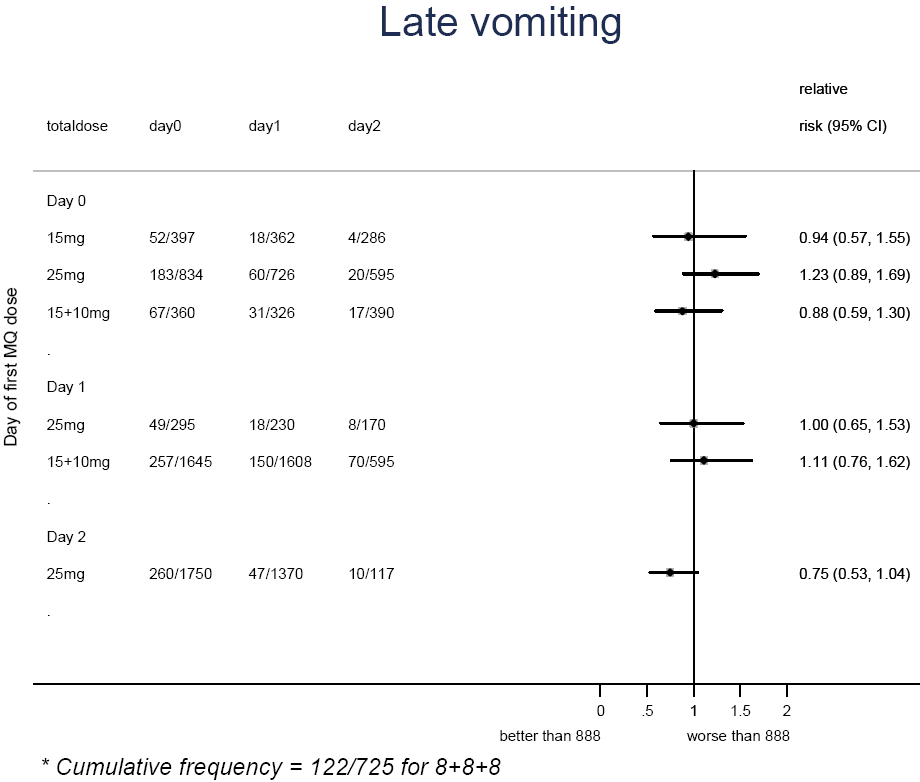

Supplement: S2 Fig — (DOCX) [file pone.0168780.s002.docx]
