# Supplementary material for: Adverse effects of mefloquine for the treatment of uncomplicated malaria in Thailand: A pooled analysis of 19, 850 individual patients
Source: PLoS One. 2017 Feb 13;12(2):e0168780. doi: 10.1371/journal.pone.0168780 (PMC5305067; doi:10.1371/journal.pone.0168780)
Supplement: S1 Table — (DOCX) [file pone.0168780.s003.docx]

| **S1 Table**: **Frequency (%) of early vomiting and relative risk (RR, unadjusted and ARR, adjusted), by day and by treatment regimen** | | | | | | | | | | | | |  |
| --- | --- | --- | --- | --- | --- | --- | --- | --- | --- | --- | --- | --- | --- |
|  | **Day 0** | | | **Day 1** | | | **Day 2** | | | **Cumulative risk** | | | |
| **Regimen** | **Freq. (%)** | **RR (CI)** | **ARR (CI)** | **Freq. (%)** | **RR (CI)** | **ARR (CI)** | **Freq. (%)** | **RR (CI)** | **ARR (CI)** | **Freq.** | **RR (CI)** | **ARR (CI)** | |
| 15 mg/kg, d0 | 35/904 (3.9) | 1.5  (0.8-2.8) | 1.4  (0.80-2.6) | 0 |  |  |  |  |  | 35/904 | 1.4  (0.8-2.4) | 0.98  (0.7-1.3) | |
| 25 mg/kg, d0 | 88/944 (9.3) | 3.7  (2.2-6.3) | 2.8  (2.1-3.8) |  |  |  |  |  |  | 88/944 | 3.3  (2.0-5.4) | 2.2  (1.7- 2.8) | |
| 25 mg/kg, d1 |  |  |  | 15/233 (6.4) | 38  (5-286) | 15.7  (4-56) |  |  |  | 15/233 | 2.3  (1.1-4.4) | 1.9  (1.6-2.3) | |
| 25 mg/kg, d2 |  |  |  |  |  |  | 62/1,674 (3.7) | 22  (3-157) | 14  (2-93) | 62/1,674 | 1.3  (0.8-2.2) | 0.90  (0.6-1.3) | |
| Split 15/10 mg/kg, start d0 | 27/447  (6) | 2.4  (1.3-4.4) | 2.4  (1.7-3.2) | 15/444 (3.4) | 19.9  (3-150) | 13  (3-49) |  |  |  | 39/449 | 3.0  (1.7-5.3) | 2.4  (2.0- 2.9) | |
| Split 15/10 mg/kg, start d1 |  |  |  | 30/1,840 (1.6) | 9.9  (1-70) | 14  (3-72) | 9/1,830 (0.5) | 2.9  (0.4-23) | 2.6  (0.3-21) | 38/1,842 | 0.7  (0.4-1.3) | 0.72  (0.49-1.1) | |
| 3-day 8+8+8 | 15/593 (2.5) |  | Ref. | 1/594 (0.17) |  | Ref. | 1/592 (0.17) |  | Ref. | 17/593 | Ref. |  | |

Early vomiting was defined as vomiting occurring within one hour of MQ treatment. The unit of analysis was an episode of vomiting for analysis by day and a patient for the cumulative risk. Risks and confidence intervals were estimated using Poisson regression with robust error variances. Adjusted analyses were stratified by year and included covariates for age, sex, baseline hematocrit, fever at admission, baseline [log] parasitaemia and the presence of vomiting at admission; more specifically, the presence of early vomiting on day 0 for day 1 risk estimates and the presence of early vomiting on day 1 for day 2 risk estimates. Cumulative risks were defined as any vomiting on any day within 1 hour of MQ treatment.
