# Supplementary material for: Adverse effects of mefloquine for the treatment of uncomplicated malaria in Thailand: A pooled analysis of 19, 850 individual patients
Source: PLoS One. 2017 Feb 13;12(2):e0168780. doi: 10.1371/journal.pone.0168780 (PMC5305067; doi:10.1371/journal.pone.0168780)
Supplement: S2 Table — (DOCX) [file pone.0168780.s004.docx]

**S2 Table: Case Reports for serious neurological adverse events**

| Case 1. A 41 year old women receiving M25 developed a paranoid psychosis with agitation and sleep disturbances on day 2. This lasted 2 weeks. She was given diazepam for 2 days and then went to a traditional healer. She had received M25 treatment two years previously without apparent problems. Her family subsequently reported she had suffered from anxiety neurosis during the previous 10 years. |
| --- |
| Case 2. A 27 year old male receiving M25 combined with single dose artesunate 4 mg/kg, had an acute paranoid psychosis on day 1, which lasted for one day. He received 1 day treatment with diazepam. It was not known if he had taken mefloquine previously. The family later reported he had a lifelong history of psychiatric disorders. |
| Case 3. A 14 year old boy with no previous history of seizures, arrhythmias or syncope, received M25, as 15 mg/kg day 0 and 10 mg/kg on day 1, as retreatment for a recrudescent infection originally treated 14 days previously with MSP. He was admitted in the MSF hospital with a single grand mal seizure on the 27th day after the last dose. He had not received quinine in the interim period. The serum mefloquine concentrations was 380 ng/ml (by high performance liquid chromatography [HPLC]). He was neurologically normal the following day and required no further treatment. |
| Case 4. A 12 year old girl was admitted with an acute psychosis which started on day 1 after treatment and lasted for 48 hours. She had received M25 as retreatment following a recrudescence of infection 14 days after treatment with M15 combined with 10 mg/kg artesunate (3.3 mg/kg given 8 hourly for 1 day). Diazepam was the only treatment given and symptoms resolved without sequelae. She had no history of previous neuropsychiatric disorders. |

Data from 2,927 patients enrolled in treatment studies between 1990 and 1994 were available for analysis of serious neuropsychiatric events following mefloquine treatment. Of them, 345 received M25 as retreatment, *i.e*. they had been treated with M15 (as monotherapy or in combination with sulfadoxine-pyrimethamine in the previous month, had a subsequent recrudescent infection, and required retreatment with M25. Half (51%) of the patients were 14 years or younger (M15 44% and M25 52%) and 61% were males (M15 66% and M25 61%). Serious neuropsychiatric reactions were reported in 0/978 M15 recipients, 2/1604 primary M25 recipients, and 2/345 patients receiving M25 as retreatment. Details of serious events on the four patients in the antimalarial treatment trials are given in the Table.
